# Novel probe based on rhodamine B and quinoline as a naked-eye colorimetric probe for dual detection of nickel and hypochlorite ions

**DOI:** 10.1038/s41598-023-44395-x

**Published:** 2023-10-09

**Authors:** Seyyed Emad Hooshmand, Behnaz Baeiszadeh, Masoumeh Mohammadnejad, Razieh Ghasemi, Farshad Darvishi, Ali Khatibi, Morteza Shiri, Faiq H. S. Hussain

**Affiliations:** 1 Department of Organic Chemistry, Faculty of Chemistry, Alzahra University, Vanak, Tehran, 1993893973 Iran; 2 Department of Analytical Chemistry, Faculty of Chemistry, Alzahra University, Vanak, Tehran, 1993893973 Iran; 3 Department of Nanotechnology, Jabir Ibn Hayyan Institute, Technical and Vocational Training Organization, Isfahan, Iran; 4 Department of Microbiology, Faculty of Biological Sciences, Alzahra University, Tehran, Iran; 5 Department of Biotechnology, Faculty of Biological Sciences, Alzahra University, Tehran, Iran; 6 Department of Medical Analysis, Faculty of Applied Science, Tishk International University-Erbil, Kurdistan Region, Iraq

**Keywords:** Chemistry, Analytical chemistry, Environmental chemistry, Green chemistry, Organic chemistry

## Abstract

This work demonstrates the design and straightforward syntheses of several novel probe-based on rhodamine B and 2-mercaptoquinoline-3-carbaldehydes as a naked-eye colorimetric probe, indicating a sensitive and selective recognition towards nickel (II) with a limit of detection 0.30 μmol L^−1^ (0.02 mg L^−1^). Further, by employing the oxidation property of hypochlorite (OCl^−^), this novel probe parallelly has been deployed to detect hypochlorite in laboratory conditions with a limit of detection of 0.19 μmol mL^−1^ and in living cells. Regarded to negligible cell toxicity toward mammalian cells, this probe has the potential to determine these analytes in in-vivo investigation and foodstuff samples.

## Introduction

In the contemporary world, the issue of “prevention is better than cure” remains a highly contentious issue in therapeutic areas. One of the pivotal aspects of achieving this goal is the rapid detection of hazardous analysts in foods, water, and living organisms. With the sustainable improvement of the cosmopolitan metropolis, the severe problem of heavy metal pollution in the air, soil, and particularly in the water environment is cause for concern^1–3^. Since the increase in water pollution could be considered an acute problem, rapid detection of heavy metal ion concentrations in drinking water is an effective remedy. One of these heavy metals that must be precisely taken into account is nickel. The transition series element nickel (Ni), which makes up around 3% of the earth's composition, is the 24th most common element on the earth. At a non-dangerous level, nickel has advantages for instance an activator of some enzyme reactions and participating in critical metabolic systems^3^. However, the ingestion of nickel beyond acceptable levels induces the inhibition of oxidative enzyme activity, adverse effects on the lungs and kidneys, skin dermatitis, gastrointestinal distress, shortness of breath, and chest pain. It is really carcinogenic, and high levels of nickel cause the reduction of nitrogen and impaired growth. Based on World Health Organization (WHO), the maximum allowable level of nickel in potable water is 0.02 mg L^−1^^4^. Hence, it is tremendously important to augment a swift and straightforward method to implement selective and sensitive recognition of nickel ions. Besides that, hypochlorite fulfills a key role in defending against the invasion of pathogens. Nevertheless, an excessive amount of hypochlorite preparation induces several diseases, namely kidney disease, arthritis, osteoarthritis, atherosclerosis, and cancer^5,6^. Consequently, rapid recognition HOCl/OCl^-^ in vitro and in vivo is really imperative. Moreover, since hypochlorite is commonly deployed as a disinfection of drinking water and household bleach, a high concentration of hypochlorite must be damaging humans and animals, causing nose irritation or arousing eye and stomach discomfort. Owing to the destructive influence of hypochlorite, it is essential to swiftly detect and monitor OCl^-^ residues^7–9^.

The sensor as well as probe fields as privileged tools for clinical diagnostics has paved the way for developing earlier diagnoses and treatments. Most popular approaches for detecting nickel rely on electrochemical methods, atomic absorption spectroscopy, and inductive coupled plasma atomic emission spectrometry (ICP-AES) for declining the interference impacts^10–12^. In spite of the great capability of the atomic techniques in selective detections, these methods have drawbacks such as their wrecking strategies, high cost as well as long-time analysis. The rational design of the naked-eye colorimetric probe as a swift and visual way culminates in detecting detrimental heavy metals and any imperative analysts^13,14^. Although many colourimetric sensors have been introduced for heavy metals such as mercury and cadmium or copper, there are very few published reports about nickel. In this line, Jiang et al.^15^ devised a novel coumarin-based colourimetric nickel sensor with high sensitivity and selectivity toward Ni^2+^ ion. Yang’s research group reported Two novel pyrazole-based chemosensors that illustrate high sensitivity and selectivity for detecting nickel^16^. Moreover, Zhang and coworkers demonstrated a colorimetric and naked-eye chemosensor based on quinoline and 2-naphthol with detect Ni^2+^ in aqueous solution with specific selectivity and high sensitivity^17^. In this area, rhodamine B reveals a wealth of opportunities for chemosensor and probe applications, and several small molecules and macromolecules-centered rhodamine B have recently been introduced as an elevated probe for mercury^18^, zinc^19^, copper^20^, lead^21^, iron^22^, picric acid^23^, and fluoride^24^. Rhodamine-centered probe universally are non-fluorescent and relatively colorless, notwithstanding they exhibit a color change to deep pink. When they are placed in an acidic solution, they demonstrate a strong fluorescence^25^. This observation is owing to protonation at the carbonyl group, and following ring-opening of its spirolactam scaffold^26^. Metals ions could cause changing the color and fluorescence by assuming a role resembling that of the hydrogen ions in acidic solvents, providing that a suitable ligand is exhibited on the spirolactam ring^27^. As a result, in the present study, in continuation of our study on quinoline chemistry^28–38^, we made an effort to enhance a novel probe based on rhodamine B and quinoline as a naked-eye colorimetric probe that could be efficiently and selectively deployed for detecting heavy metals, namely nickel and also hypochlorite. In addition to that, the chemodosimeter of the novel rhodamine-based probe for analyte with high sensitivity showed a drastic color change from colorless to deep pink after the addition of the analyte^39^. To the best of our knowledge, this is the first time that a dual colourimetric probe for nickel and hypochlorite ions is devised.

## Experimental

### Reagents and instruments

The chemicals such as Nickel chloride, rhodamine B, and hydrazine were prepared by Aldrich. The solvents were analytical grade and purchased from Merck. Deionized water was used for the dilution of the solutions. The absorbance measurement was carried out by double-beam UV–Vis spectrophotometer using a 1 cm quartz cell (Perkin-Elmer, Lambda 35, USA). The fluorescence measurement was carried out by a Pl spectrophotometer using a 1 cm quartz cell (Cary Eclipse Agilent G980A). ^1^H-NMR and ^13^CNMR spectra were recorded on Bruker AQS AVANCE 500, 400, and 300 MHz spectrometers respectively, using TMS as an internal standard and DMSO-d6 and CDCl_3_ as solvent.

### General synthesis of probe based on rhodamine B and quinoline

In a 50 ml flask, 1 mmol (0.203 g) of the 2-mercapto-6-methyl quinoline-3-carbaldehyde compound and 1 mmol (0.465 g) rhodamine B hydrazide under reflux conditions and nitrogen atmosphere in methanol were mixed by a magnetic stirrer. The progress of the reaction was monitored by TLC in the mixture of n-hexane and ethyl acetate in a ratio of 6:4, the reaction was completed after 24 h. The resulting precipitates were purified by washing with methanol and the final product **7a** was obtained as a yellow precipitate with a yield of 85%.

## Results and discussion

**Figure 1:**
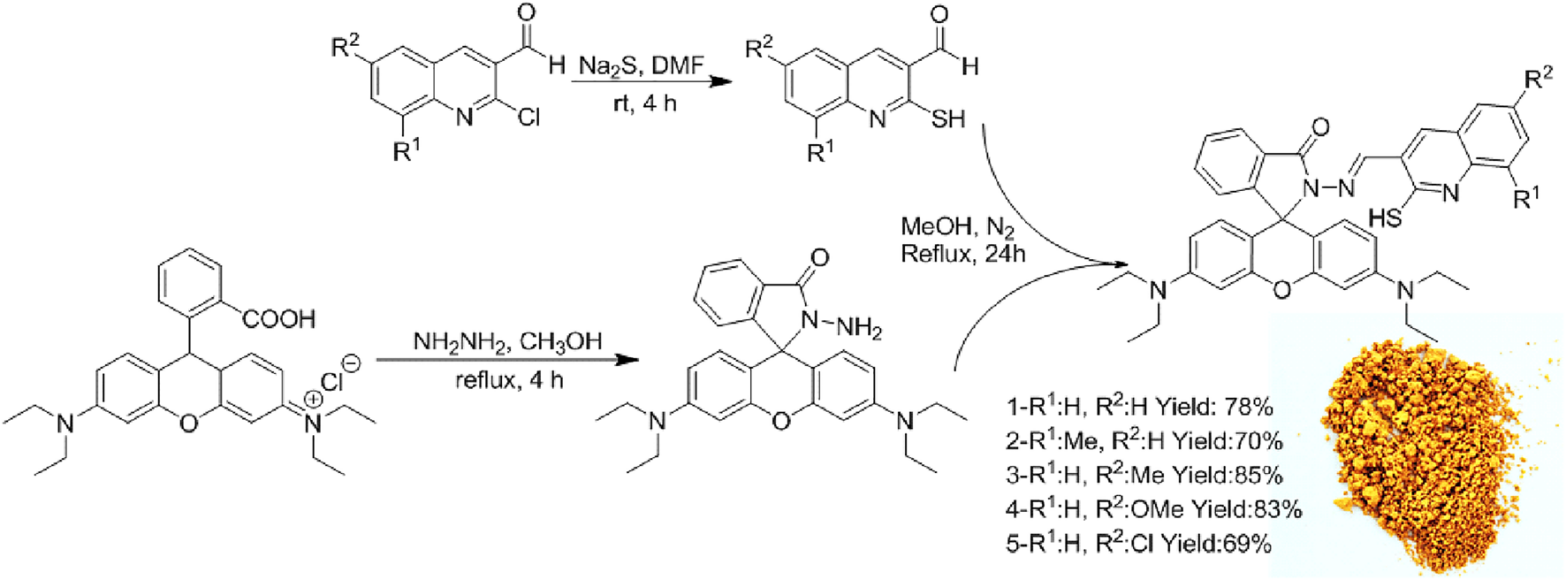
Synthesis of novel rhodamine-based probe.

After facile synthesis of rhodamine hydrazide^20^ and 2-mercaptoquinoline-3-carbaldehydes^40^ based on previous reports, we commenced our work by the addition of rhodamine hydrazide with 2-mercaptoquinoline-3-carbaldehydes in MeOH under refluxing conditions yields the novel rhodamine-based probe with appropriate yield (Fig. 1). Next, the synthetic rhodamine-based probe was characterized by FTIR, ^1^H-NMR, and ^13^C-NMR.

**Figure 2:**
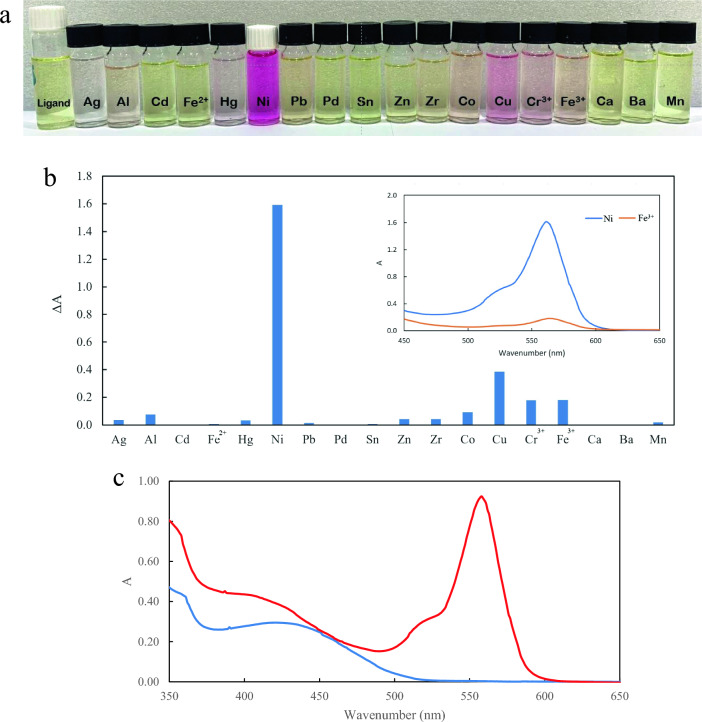
(**a**) Photographic images of colorimetric detection of various metals in water through simple naked-eye analysis. (**b**) Absorbance changes of the novel rhodamine-based probe in the presence of various metal ions and for example UV–visible spectra in the presence of Ni^2+^ and Fe^3+^ (inserted). (**c**) The UV–visible absorption spectra of the probe in the absence (blue) and presence (red) of Ni^2+^.

The interaction between a synthesized rhodamine-based probe and several metal ions (Fig. 2a) was investigated using the same settings. It was clear that the addition of an extensive diversity of environmentally metal ions namely Cu^2+^, Pb^2+^, Zr^2+^, Zn^2+^, Ag^+^, Al^3+^, Hg^2+^, Co^2+^, Mn^2+^, Cr^3+^, Sn^2+^, Fe^3+^, Cd^2+^, Al^3+^, Pd^3+^, Fe^2+^, Ca^2+^, Ba^2+^, Mn^2+^, Ni^2+^, did not have as a quite much effect on the probe as Ni^2+^ (Fig. 2b). The outcome supported the high selectivity of the novel probe which is considered a pivotal characteristic of an ion-selective probe. Figure 2c depicts the UV–visible absorption spectra of this new probe in the absence and presence of Ni^2+^. Ni^2+^ could chelate with the probe, causing a ring-opening reaction of its spirolactam scaffold, and then coordinate with carbonyl oxygen, nitrogen atoms of two imine groups, and quinoline nitrogen when added to the probe solution^25^. It resulted in a blue shift in the absorption spectrum, the formation of a new band at 560 nm, and a steady rise in absorbance with increasing Ni^2+^ concentration. Consequently, the probe solution changed from yellow to deep pink.

**Figure 3:**
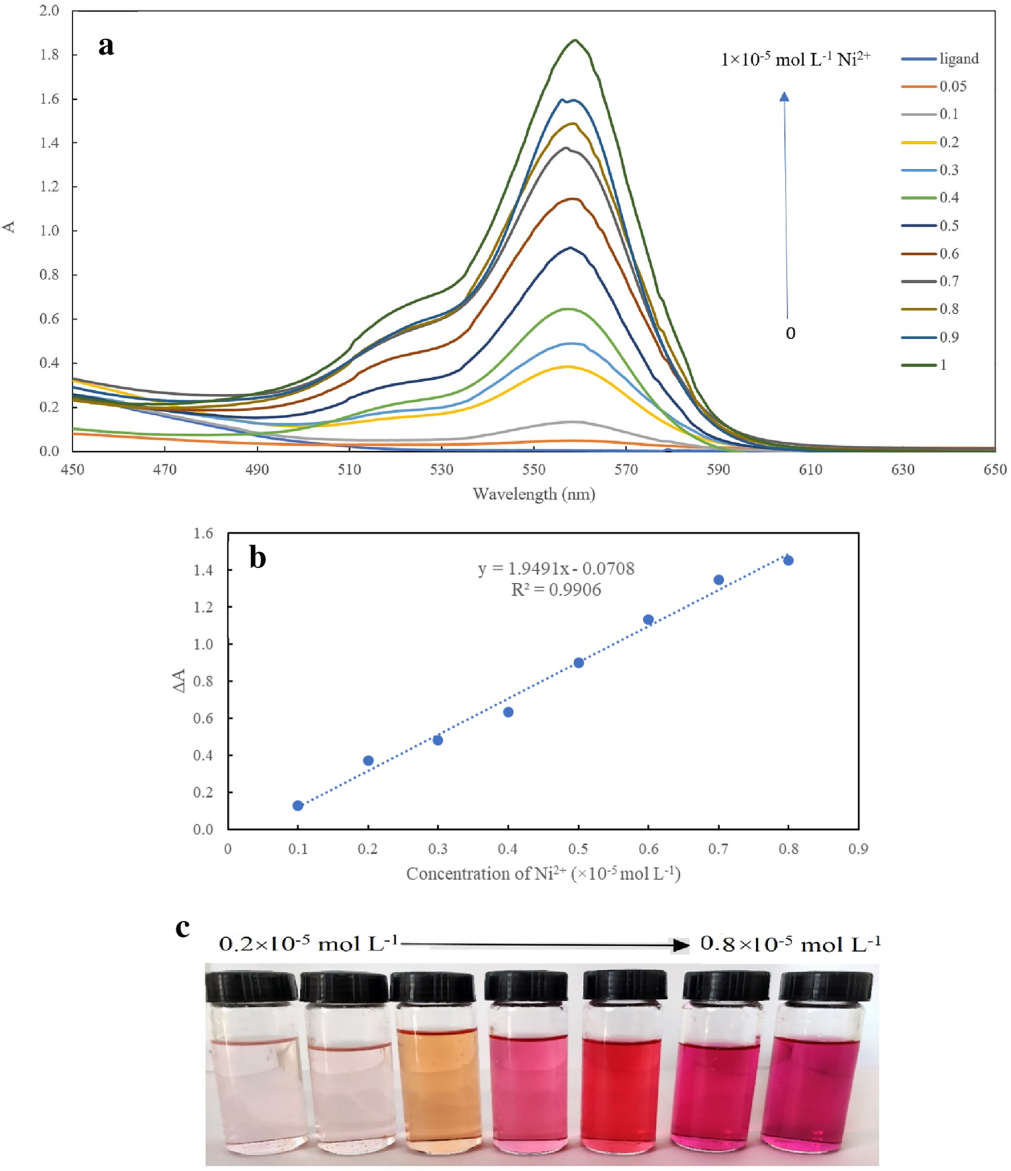
(**a**) Absorption spectra of rhodamine-based probe (5 × 10^–5^ M) with the addition of Ni (0.05, 0.1, 0.2, 0.3, 0.4, 0.5, 0.6, 0.7, 0.8, 0.9, 1 × 10^–5^ M); (**b**) linearity of absorbance intensity of rhodamine-based probe with Ni from 0.1 to 0.8 × 10^-5^ M. (**c**) Photographic images of colorimetric detection of nickel (0.2–0.8 × 10^–5^ mol L^−1^) in water through simple naked-eye analysis.

For investigation of the interaction of Ni^2+^ with novel rhodamine and quinoline-based probe, various concentrations of this element solution were added to the solution and absorbance spectra were recorded up to 30 min. By increasing the concentration of Ni, the new peak at 560 nm was observed, and the intensity increased steadily. The absorption spectra are illustrated in Fig. 3a. As a matter of fact, the intensity of the absorbance at 560 nm pertaining to the addition of Ni^2+^ concentration drastically, and the calibration curve was plotted based on the absorbance increasing at 560 nm in the presence of Ni^2+^ (Fig. 3b) in the range of 0.1–0.8 × 10^–5^ mol L^−1^ with a detection limit of 0.03 × 10^–5^ mol L^−1^. The color of the solution swiftly changed from yellow to deep pink which can be rapidly diagnosed by the naked eye. A color gradient from yellow to deep pink was obviously perceived upon increasing the concentration of nickel which permitted a semiquantitative detection of the analyte in water by swift and facile visual analysis (Fig. 3c). Evidently, the color intensity became stronger with increasing the concentration of Ni^2+^.

**Figure 4:**
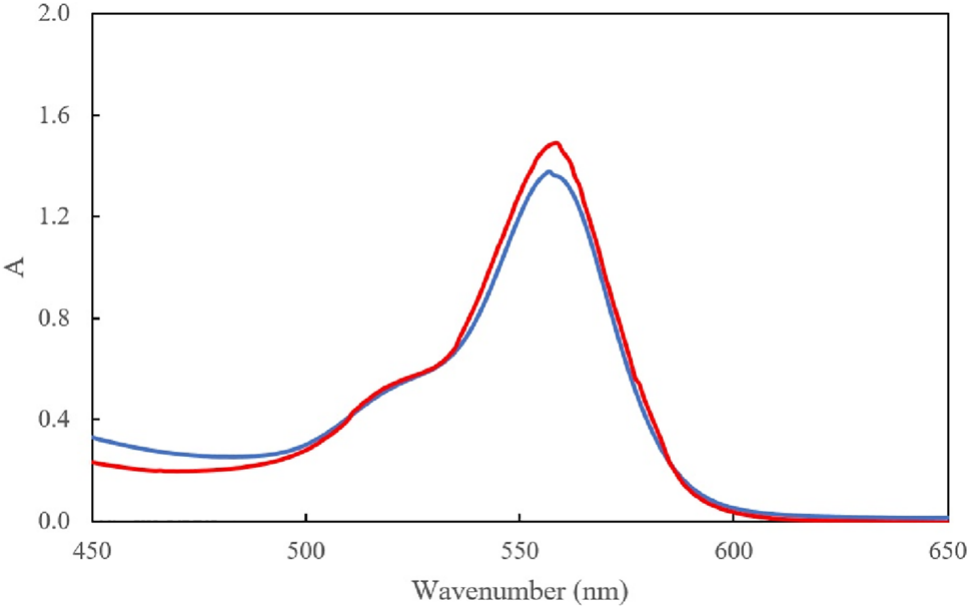
Absorption spectra of rhodamine-based probe with Ni^2+^ (blue spectrum) and the spectra of this solution (probe and Ni^2+^) in the presence of Cu^2+^.

To study the selectivity of the synthesized probe, the effect of different cations and anions such as Cu^2+^, Pb^2+^, Zr^2+^, Zn^2+^, Ag^+^, Al^3+^, Hg^2+^, Co^2+^, Mn^2+^, Cr^3+^, Sn^2+^, Fe^3+^, Cd^2+^, Al^3+^, Pd^3+^, Fe^2+^, Ca^2+^, Ba^2+^, Mn^2+^, Cl^−^, NO_3_^-^, PO_4_^3−^, S^2−^ and SO_4_^2−^ on determination of Ni^2+^ was investigated. Different concentration of these ions were added to the probe-Ni solutions and the absorption spectra of rhodamine-based probe were recorded. The tolerance limit of an ion was taken as the maximum amount of the ion causing an error not greater than ± 5%. A 100-fold excess of these cation and anions did not interfere on the determination of Ni^2+^ and the major interference was Cu^2+^ (Fig. 4). The interfering effect of Cu^2+^, was successfully removed by addition of dilute sulfide solution into the sample solution.

**Figure 5:**
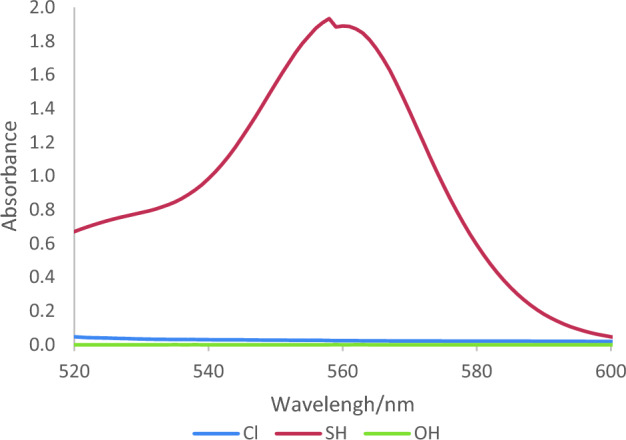
Effect of substitutions of position 2 of quinoline-3-carbaldehydes in performance of novel rhodamine-based probes.

**Figure 6:**
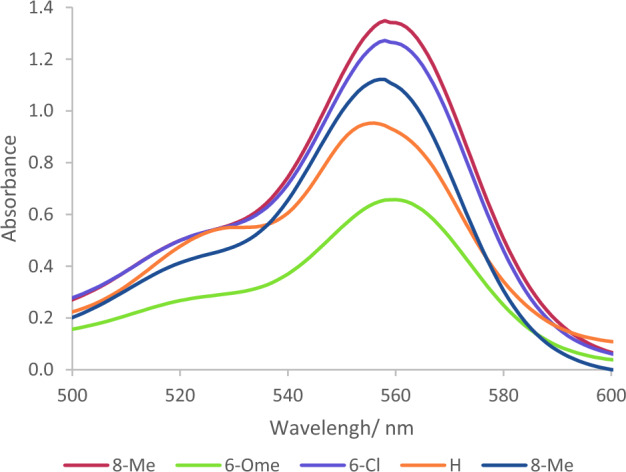
Effect of substitutions on 2-mercaptoquinoline-3-carbaldehydes in performance of novel rhodamine-based probes.

Moreover, the role of functional groups in this naked-eye colorimetric probe was perused. Accordingly, in addition to 2-mercaptoquinoline-3-carbaldehydes, 2-chloroquinoline-3-carbaldehyde and 2-hydroxyquinoline-3-carbaldehyde have been deployed in this project. As expected, on account of the appropriate affinity of mercaptans with such metals, a rhodamine-based probe prepared by 2-mercaptoquinoline-3-carbaldehydes revealed elevated interaction with nickel in comparison with hydroxy and chloro derivatives. (Fig. 5) Furthermore, different 2-mercaptoquinoline-3-carbaldehydes were used in probe scaffolds and subsequently the interaction of probe-encompassing substitutions namely methyl, methoxy or chlorine with Ni^2+^ were investigated. The outcome depicted the elevated tendency of the probe prepared by 2-mercapto-6-methylquinoline-3-carbaldehyde to the analyte than the other derivatives (Fig. 6).

**Figure 7:**
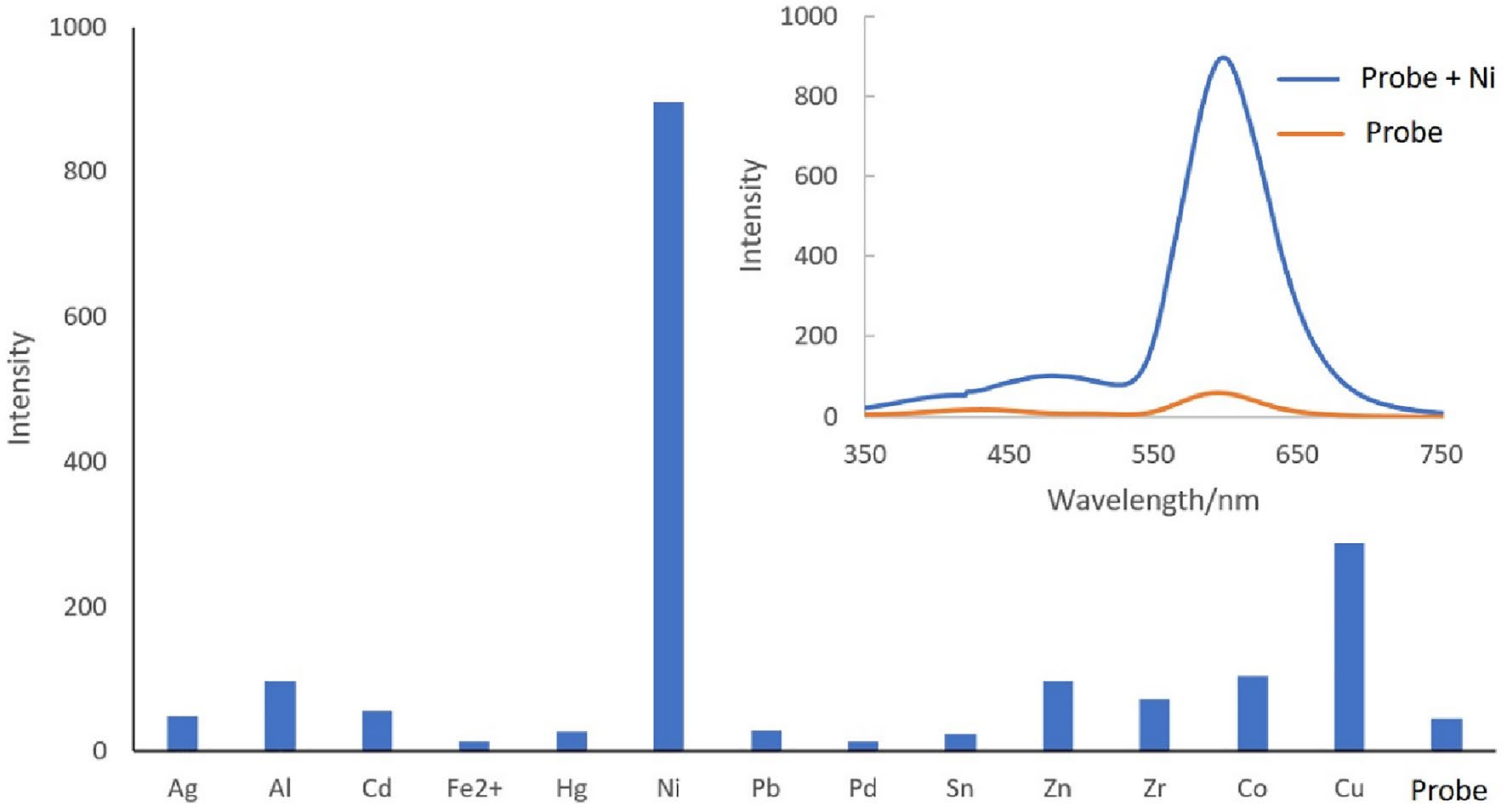
Fluorescence intensity of probe in the presence of different metal ions (inset:fluorescence spectra of probe and probe-Ni).

In addition, based on the structure of the synthesized component, its fluorescence spectra were recorded in the presence of different ions with λ_ex_ = 310 nm (Fig. 7 inset). The fluorescence spectrum of the synthesized component shows a very low fluorescence intensity in λ_em_ = 590 nm that is dramatically increase in the presence of Ni^2+^ compared to some other metal ions. These fluorescence results confirm the absorbance changes of probe by Ni^2+^.

### Detection of hypochlorite

**Figure 8:**
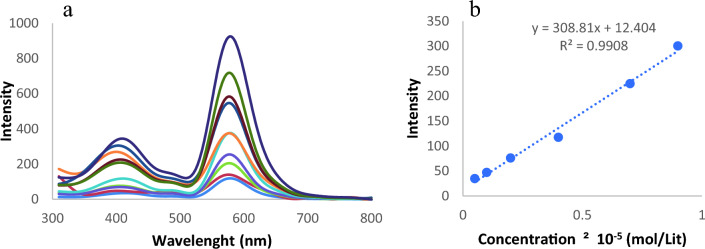
(**a**) Fluorescence spectra of probe rhodamine B-based probe (5 × 10^–5^ M) with the addition of ClO^−^ (0.05, 0.1, 0.2, 0.3, 0.4, 0.5, 0.6, 0.7, 0.8, 0.9, 1 × 10^-5^ M). (**b**) Linearity of fluorescence intensity of rhodamine B-based probe with ClO^-^.

To study the interaction of the probe by ClO^−^, the fluorescence properties of the synthesized component were recorded. The fluorescent titration profiles of the probe (5 × 10^–5^) with ClO^−^ (0.05–1 × 10^–5^ M) are illustrated in Fig. 8a. As the concentration of ClO^−^ was titrated into the probe, there was a noticeable fluorescence amplification at λ_em_ = 410 and 590 nm (with λ_ex_ = 310 nm). The ring-opening rhodamine acid caused by the interaction of the probe rhodamine B-based probe with ClO^-^ is another possible explanation for the outcomes. Additionally, the probe rhodamine B-based probe exhibits remarkable linearity between fluorescence intensity (at λ_em_ = 410 nm) and ClO^-^ concentration across the range of 0.05–0.9 × 10^–5^ M (Fig. 8b). The detection limit was calculated at 0.19 µM, which also demonstrated a highly sensitive characteristic. This outcome suggests that the probe may be used to quantitatively detect HOCl/ClO^−^.

The observed changes in the absorption and emission colours of the probe upon the addition of OCl^−^ can be attributed to the opening of the spirolactam ring in the rhodamine motif. In most cases, the process of ring opening in cations is facilitated by the chelation of cations with the rhodamine-linked derivative. This leads to the creation of a chelation-enhanced colorimetric and fluorescence probe, which can detect various metal ions. The probe operates by converting the spirolactam form of the rhodamine dye (which is colorless and nonfluorescent) to the ring-open amide form (which is deep pink colored and exhibits strong fluorescence)^41–44^.

To study the effect of different anions on the ClO^−^ determination, different anions such as Cl^−^, NO_3_^−^, PO_4_^3−^, SO_4_^2−^ and S^2−^ was studied in selectivity of the synthesized probe. Based on the results, these anions do not show effect on the signal up to 1000 order of ClO^−^ concentration.

### The novel probe function in living cells

**Figure 9:**
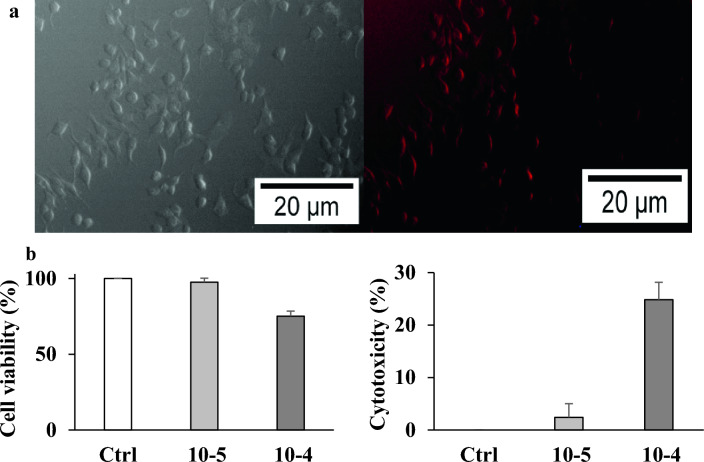
(**a**) Light and fluorescence images of MCF7 cells treated with the rhodamine B-based probe probe. (**b**) Cell viability and cytotoxicity effects of two concentrations (10^–4^ and 10^–5^ M) of the probe on MCF7 cells after 24 h.

The yeast cells of *Saccharomyces cerevisiae* ATCC9763 were treated with two different concentrations (10^–4^ and 10^–5^ M) of the probe. The probe was not observed inside yeast cells by fluorescence microscopy. To determine the function of the novel probe in mammalian cells and its cell viability and cytotoxicity, the breast cancer MCF7 cells were treated with two concentrations (10^–4^ and 10^–5^ M) of the probe. Images of fluorescence microscopy show that the probe has entered the cells. Therefore, a probe rhodamine B-based probe can be used for fluorescence microscopy imaging of MCF7 cells (Fig. 9a).

As shown in Fig. 9b, the cell viability and cell toxicity were obtained at 97.59% and 2.4%, and 75.16% and 24.83% for 10^–5^ and 10^–4^ M concentration respectively after 24 h via MTT assay. The results show that the cell viability decreases when the concentration of the probe increases, but this change is not very significant, and the probe has no cytotoxicity for MCF7 cells.

## Conclusion

Here, we have devised a highly efficient novel probe based on rhodamine B and 2-mercaptoquinoline-3-carbaldehydes for dual sensing heavy metals and hypochlorite. The probe depicted a significant color change from colorless to pink and fluorescence improvement upon the addition of nickel and ClO^−^ ions. The probe moreover had a low limit of detection (0.3 μmol mL^−1^ for nickel and 0.19 μmol mL^−1^ for ClO^-^ ions). Considering negligible toxicity toward mammalian cells and the successful application of this novel probe as an imaging agent for endogenous hypochlorite in living cells, this probe-centered rhodamine could promisingly be used in industrial applications and foodstuff samples.

### Supplementary Information

Supplementary Information.

## Data Availability

All data generated or analyzed during this study are included in this published article [and its Supplementary Information file].
