# Supplementary material for: Novel probe based on rhodamine B and quinoline as a naked-eye colorimetric probe for dual detection of nickel and hypochlorite ions
Source: Sci Rep. 2023 Oct 9;13:17038. doi: 10.1038/s41598-023-44395-x (PMC10562415; doi:10.1038/s41598-023-44395-x)
Supplement: Supplementary file 1 — Supplementary Information. [file 41598_2023_44395_MOESM1_ESM.docx]

**Novel probe** **based on rhodamine B and quinoline as a naked-eye colorimetric sensor for dual sensing of heavy metals and hypochlorite as a biosensing and bioimaging agent**

Seyyed Emad Hooshmand,^a^ Behnaz Baeiszadeh,^a^ Masoumeh Mohammadnejad,^a^ Razieh Ghasemi,^b^ Farshad Darvishi,^c^ Ali Khatibi,^d^ Morteza Shiri,^a,*^ Faiq H. S. Hussain^e,*^

^a^Department of Chemistry, Faculty of Physics and Chemistry, Alzahra University, Vanak, Tehran 1993893973, Iran. E-mail: [mshiri@alzahra.ac.ir](mailto:mshiri@alzahra.ac.ir)

^b^Department of Nanotechnology, Jabir Ibn Hayyan Institute, Technical and Vocational Training Organization, Isfahan, Iran.

^c^Department of Microbiology, Faculty of Biological Sciences, Alzahra University, Tehran, Iran.

^d^Department of Biotechnology, Faculty of Biological Sciences, Alzahra University, Tehran, Iran.

^e^Department of Medical Analysis, Faculty of Applied Science, Tishk International University - Erbil, Kurdistan Region, Iraq.

**Contents** **Pages** Experimental section (general procedure and characterization data for all compounds) 2-6

Copies of ^1^H and ^13^C NMR spectra for all products 7-18

1. Experimental Section

**Reagents and instruments**

The chemicals such as Nickel chloride, rhodamine B and hydrazine were prepared by Aldrich. The solvents were analytical grade and purchased from Merck. Deionized water was used for the dilution of the solutions. The absorbance measurement was carried out by double-beam UV-Vis spectrophotometer using a1 cm quartz cell (Perkin-Elmer, Lambda 35, USA).

The fluorescence measurement was carried out by Pl spectrophotometer using a 1 cm quartz cell (Cary Eclipse Agilent G980A).

^1^H-NMR, and ^13^CNMR spectra were recorded on Bruker AQS AVANCE 500, 400 and 300 MHz spectrometers respectively, using TMS as an internal standard and DMSO-d6 and CDCl_3_ as solvent.

**General synthesis of 2-mercapto-6-methylquinoline-3-carbaldehyde and 2-hydroxy-6-methylquinoline-3-carbaldehyde**

In a 25 ml flask, 1 mmol of 2-chloroquinoline-3-carbaldehyde derivatives (1), and 2 mmol of sodium sulfide were combined with 5 ml of DMF as a solvent. The reaction mixture was stirred at room temperature under magnetic stirring for 4 hours. The progress of the reaction was monitored by TLC. After 4 hours, the reaction mixture was added to a beaker containing water and ice along with 5 ml of acetic acid. The final product in the form of a yellow precipitate was smoothed and washed with water.(Figure 1)

**Figure 1.** Synthesis of 2-mercapto-6-methylquinoline-3-carbaldehyde

In a 25 ml flask, the amount of 1 mmol (0.205 g) of 2-chloro-6-methylquinoline-3-carbaldehyde compound (a1) along with 3 ml of 4N hydrochloric acid was placed under reflux and magnetic stirrer conditions. The progress of the reaction was monitored by TLC. After 2 hours, the reaction was complete. The resulting white precipitate was filtered and washed with water. The melting point of this compound is 265-267 ℃.(Figure 2)

**Figure 2.** Synthesis of 2-hydroxy-6-methylquinoline-3-carbaldehyde

**Typical synthesis of** **rhodamine B hydrazide**

In a 25 ml flask, 1 mmol (0.479 g) of rhodamine B was combined with 5 ml of methanol as a solvent. The reaction mixture was subjected to reflux and a magnetic stirrer for 4 hours. During the reaction, the amount of 144 mmol (7 ml) of hydrazine hydrate was added drop by drop to the reaction at regular intervals for 4 hours. The progress of the reaction was observed by changing the color of the mixture from purple to orange and the formation of sediment. The resulting sediments were separated from the reaction mixture, and the excess amount of hydrazine hydrate was removed by washing with water. The final product was obtained as a white precipitate with an efficiency of 87%. (Figure 3)

**Figure 3.** Synthesis of rhodamine B hydrazide

**General synthesis of** **sensors based on rhodamine B and quinoline**

In a 25 ml flask, the amount of 1 mmol (0.205 g) of 2-chloro-6-methylquinoline-3-carbaldehyde (1a) and 1 mmol of rhodamine B hydrazide (4) under reflux conditions and methanol solvent - The magnetic stirrer was mixed. The progress of the reaction was monitored by TLC in n-hexane and ethyl acetate in a ratio of 8:2. The reaction was completed after 24 hours, and the resulting yellow precipitate was purified by washing with methanol. The yield of the product was calculated to be 90%. (Figure 4)

**Figure 4.** Synthesis of sensors based on rhodamine B and quinoline

In a 50 ml flask, 1 mmol (0.187 g) of 2-hydroxy-6-methylquinoline-3-carbaldehyde compound (3) and 1 mmol (0.465 g) of rhodamine B hydrazide (4) under the reflux conditions and methanol solvent were mixed by a magnetic stirrer. The progress of the reaction was monitored by TLC in n-hexane and ethyl acetate in a ratio of 8:2 and the reaction was completed after 24 hours. The resulting precipitates were purified by washing with methanol, and the final product (6) was obtained in the form of a white precipitate with an efficiency of 81%, which was identified by examining the ^1^H NMR and ^13^C NMR spectra. (Figure 5)

**Figure 5.** Synthesis of product 6

In a 50 ml flask, 1 mmol (0.203 g) of 2-mercapto-6-methylquinoline-3-carbaldehyde compound (2a) and 1 mmol (0.465 g) rhodamine B hydrazide (4) under reflux conditions and nitrogen atmosphere and methanol solvent were mixed by a magnetic stirrer. The progress of the reaction was monitored by TLC in the solvent of n-hexane and ethyl acetate in a ratio of 6:4 and the reaction was completed after 24 hours. The resulting precipitates were purified by washing with methanol, and the final product a7 was obtained as a yellow precipitate with a yield of 85%. (Figure 6)

**Figure 6.** Synthesis of sensor 7a

**Preparation of solutions for colorimetric and Fluorescence study**

Stock solutions of Ni^2+^ solution (10^-3^ mol L^-1^) were prepared by dissolving appropriate amount of Nickel chloride in deionized water. RB-CQC stock solution (1.0× 10^-4^ mol L^-1^) was prepared in EtOH solution. In detection, RB-CQC was diluted to 5.0× 10-5 mol L^-1^ by EeOH. each time 2.0 mL of 5.0× 10^-5^ mol L^-1^ RB-CQC was placed into a 1.0 cm path-length cuvette, and the required quantity of Ni^2+^ solution was added by a micro-pipette. 30 min later the UV–Vis and Pl spectra were measured. All the measurements were taken at room temperature. For the interference detection, solutions of Pb^2+^, Zn^2+^, Cd^2+^, Fe^3+^, Hg^2+^, Co^2+^, Fe^3+^, Zr^3+^, Ag^+^, Cu^2+^, Cr^3+^, Al^3+^, Pd^3+^, Ni^2+^ from their nitrate or chloride were used.

**Spectral data and physical properties**

**(E)-3',6'-Bis(diethylamino)-2-(((2-hydroxy-6-methylquinolin-3-yl)methylene) amino)spiro [isoindoli -ne -1,9'-xanthen]-3-one (6):**

(81%) as a Yellow Powder, M.P: 198-200 ℃. FT-IR: νmax = 3447, 2970, 1700, 1633, 1545, 1466, 1221, 1091, 757 cm^-1^. ^1^H NMR (500 MHz, CDCl_3_): δ 8.69 (1H, s, OH), 8.52 (1H, s, N=C-H), 8.06 (1H, d, *J* = 6.5 Hz, CH-Ar), 7.77 (1H, d, *J* = 8.5 Hz, CH-Ar), 7.56 (1H, s, CH-Ar), 7.53 (1H, s, CH-Ar), 7.51 (1H, d, *J* = 5.5 Hz, CH-Ar), 7.48 (1H, d, *J* = 4.7 Hz, CH-Ar), 7.46 (1H, s, CH-Ar), 7.132 (1H, d, *J*  = 7.0 Hz, CH-Ar), 6.55 (1H, d, *J* = 8.5 Hz, CH-Ar), 6.47 (2H, s, CH-Ar), 6.42 (1H, s, CH-Ar), 6.27 (2H, d, *J* = 8.0 Hz, CH-Ar), 3.31-3.34 (8H, m, 4CH_2_), 2.48 (3H, s, CH_3_), 1.131-1.155 (12H, t, 4CH_3_) ppm.

^13^C NMR (128 MHz, CDCl_3_): δ 165.4, 152.8, 149.0, 146.2, 140.1, 137.2, 134.8, 133.9, 133.3, 128.4, 128.1, 128.0, 127.7, 127.2, 126.8, 123.9, 123.6, 108.2, 104.6, 98.0, 65.7, 44.4, 21.6, 12.6 ppm.

**(E)-3',6'-Bis(diethylamino)-2-(((2-mercapto-6-methylquinolin-3 yl) methylene)**

**amino) spiro [isoindoli -ne -1,9'-xanthen]-3-one (7a)**

(85%) as a Yellow powder, M.P: 172-174 ℃. FT-IR: νmax = 2969, 1694, 1615, 1545, 1466, 1222, 1089, 757 cm^1^.

^1^H NMR (300 MHz, CDCl_3_): δ 11.47 (1H, s, SH), 9.08 (1H, s, N=C-H), 8.13 (1H, s, CH-Ar), 7.96 (1H, s, CH-Ar), 7.43 (2H, s, CH-Ar), 7.31 (2H, s, CH-Ar), 7.22 (2H, t, *J*  = 17.7 Hz, CH-Ar), 7.04 (1H, s, CH-Ar), 6.45 (3H, d, *J*  = 15 Hz, CH-Ar), 6.12 (2H, s, CH-Ar), 3.24 (8H, s, 4CH_2_) 2.31 (3H, s, CH_3_), 1.08 (12H, s, 4CH_3_) ppm . ^13^C NMR (128 MHz, CDCl_3_): δ 168.3, 157.5, 157.0, 136.7, 132.1, 130.9, 130.1, 129.3, 128.4, 128.2, 127.4, 125.3, 122.9, 120.9, 114.2, 110.2, 109.2, 108.8, 105.1, 66.8, 55.2, 39.0, 32.2, 24.6 ppm.

**(E)-3',6'-Bis(diethylamino)-2-(((2-mercapto-8-methylquinolin-3 yl) methylene)**

**amino) spiro [isoindoli -ne -1,9'-xanthen]-3-one (7b)**

(70%) Yellow Powder, M.P: 172-174 ℃. FT-IR: νmax = 2969, 1694, 1700, 1634, 1544, 1468, 1265, 1117, 755 cm^-1^. ^1^H NMR (500 MHz, CDCl_3_): δ 10.21 (1H, s, SH), 8.95 (1H, s, N=C-H), 8.33 (1H, s, CH-Ar), 8.04 (1H, d, *J* = 7.5 Hz, CH-Ar), 7.52 (1H, d, *J* = 8 Hz, CH-Ar), 7.48 (1H, d, *J* = 6 Hz, CH-Ar), 7.45 (1H, d, *J* = 7 Hz, CH-Ar), 7.31 (1H, t, *J* = 16.5 Hz, CH-Ar), 7.17 (1H, t, *J* = 15 Hz, CH-Ar), 7.12 (1H, d, *J* = 7.5 Hz, CH-Ar), 6.54 (2H, d, *J* = 9 Hz, CH-Ar), 6.51 (2H, s, CH-Ar), 6.26 (2H, d, *J* = 7.5 Hz, CH-Ar), 3.25-3.39 (8H, m, 4CH_2_) 2.43 (3H, s, CH_3_), 1.15 (12H, t, *J* = 15 Hz, 4CH_3_) ppm .

^13^C NMR (100 MHz, DMSO): δ 180.7, 164.5, 153.0, 152.5, 148.2, 139.8, 134.6, 133.1, 132.5, 130.9, 129.4, 129.3, 128.2, 127.7, 125.0, 124.2, 124.2, 123.6, 122.4, 108.6, 105.2, 98.3, 65.4, 44.2, 15.7, 12.9, 12.9, 12.8.

**(E)-3',6'-Bis(diethylamino)-2-(((2-mercapto-6-methoxyquinolin-3 yl)methylene)**

**amino) spiro [isoindoli -ne -1,9'-xanthen]-3-one** **(7c)**

(83%) as a Yellow powder, M.P:178-180 ℃. FT-IR: νmax = 2966, 1698, 1651, 1459, 1235, 1117, 756 cm^-1^.

^1^H NMR (500 MHz, CDCl_3_): δ 11.83 (1H, s, SH), 9.03 (1H, s, N=C-H), 8.19 (1H, s, CH-Ar), 7.96 (1H, d, *J* = 11.5 Hz, CH-Ar), 7.364-7.451 (4H, m, CH-Ar), 7.04 (2H, d, *J* = 11.5 Hz, CH-Ar), 6.90 (1H, s, CH-Ar), 6.47 (2H, d, *J* = 15 Hz, CH-Ar), 6.42 (1H, s, CH-Ar), 3.75 (3H, s, OCH_3_), 3.23 (8H, d, *J* = 11.5 Hz, 4CH_2_), 1.06 (12H, t, *J* = 23.5 Hz, 4CH_3_) ppm.

^13^C NMR (128 MHz, DMSO): δ 165.6, 156.4, 153.9, 153.1, 152.8, 149.0, 143.8, 134.5, 133.8, 131.5, 128.1, 127.8, 123.9, 123.6, 122.5, 117.2, 108.2, 105.1, 98.5, 98.0, 66.0, 58.4, 55.6, 44.4, 18.4, 12.6 ppm.

**(E)-3',6'-Bis(diethylamino)-2-(((6-chloro-2-mercaptoquinolin-3 yl)methylene)**

**amino) spiro [isoindoli -ne -1,9'-xanthen]-3-one (7d)**

(69%) as a Yellow Powder, M.P: 175-177 ℃. FT-IR: νmax = 2925, 1699, 1651, 1684, 1581, 1458, 1218, 1118, 757 cm^-1^ . ^1^H NMR (500 MHz, CDCl_3_): δ 11.36 (1H, s, SH), 9.08 (1H, s, N=C-H), 8.17 (1H, s, CH-Ar), 8.03 (1H, d, *J* = 7 Hz, CH-Ar), 7.58 (1H, s, CH-Ar), 7.52 (1H, t, *J* = 7 Hz, CH-Ar), 7.499-7.487 (1H, t, CH-Ar), 7.20 (2H, d, *J* = 9 Hz, CH-Ar), 7.12 (1H, d, *J* = 7.5 Hz, CH-Ar), 6.52 (2H, d, *J* = 9 Hz, CH-Ar), 6.49 (2H, s, CH-Ar), 6.26 (2H, d, *J* = 8.5 Hz, CH-Ar), 3.29-3.34 (8H, m, 4CH_2_), 1.14 (12H, t, *J* = 14 Hz, 4CH_3_) ppm .

^13^C NMR (100 MHz, DMSO): δ 192.1, 180.9, 164.6, 152.8, 143.3, 140.2, 138.5, 134.8, 134.5, 133.9, 132.3, 129.8, 129.3, 128.9, 128.2, 127.8, 124.2, 123.7, 123.6, 123.3, 118.7, 118.2, 44.3, 12.7 ppm.

**(E)-3',6'-Bis(diethylamino)-2-(((2-mercaptoquinolin-3-yl)methylene)amino)spiro [isoindoli -ne -1,9'-xanthen]-3-one (7e)**

(78%) as a Yellow Powder, M.P: 216-218 ℃. FT-IR: νmax = 2967, 1697, 1633, 1548, 1466, 1219, 1076, 758 cm^-1^.

^1^H NMR (300 MHz, DMSO): δ 13.90 (1H, s, OH), 9.32 (1H, s, N=C-H), 7.48-8.05 (7H, m, CH-Ar), 7.00 (1H, s, CH-Ar), 6.35 (7H, s, CH-Ar), 3.32 (8H, s, 4CH_2_), 1.08 (12H, s, 4CH_3_) ppm .

^13^C NMR (75 MHz, DMSO): δ 165.8, 153.5, 152.4, 148.5, 130.1, 128.2, 124.9, 124.0, 123.5, 122.6, 108.2, 105.9, 97.9, 65.2, 44.1, 40.8, 40.5, 40.2, 39.7, 39.1, 12.9 ppm .

**3. Original copies of NMR spectra**


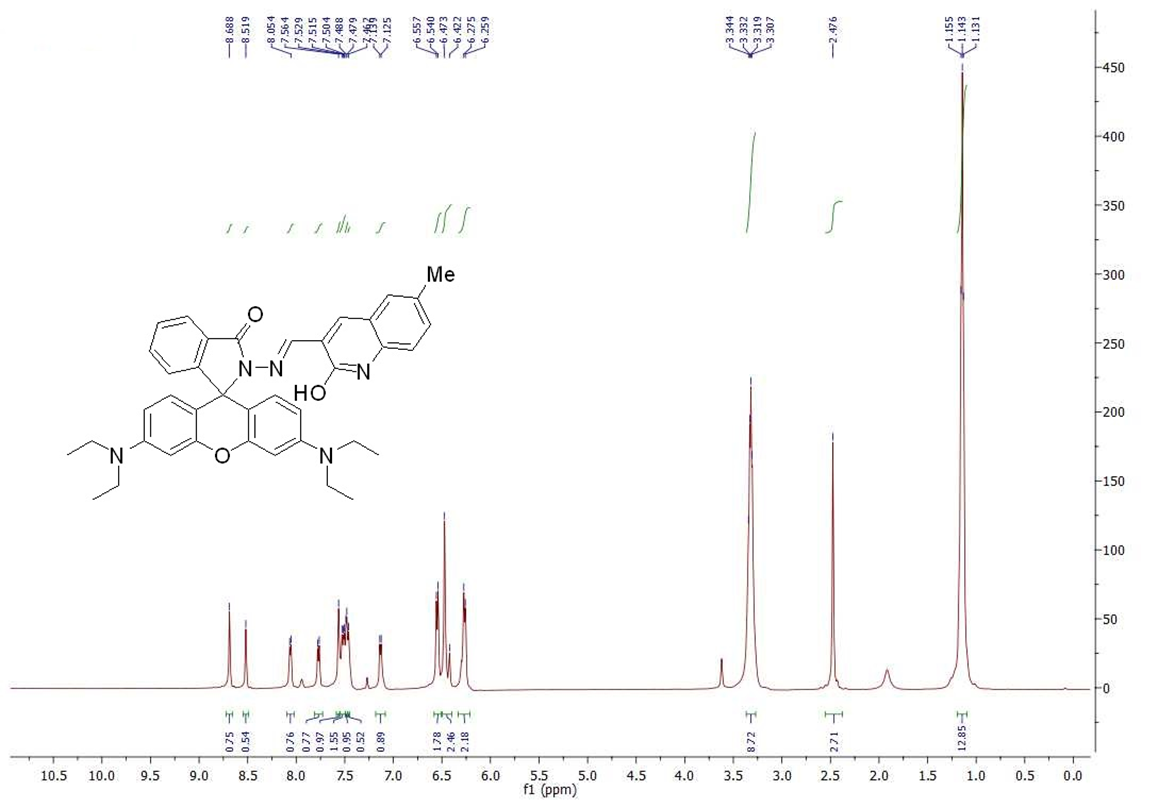


**Figure 7.** ^1^H NMR spectrum for 6


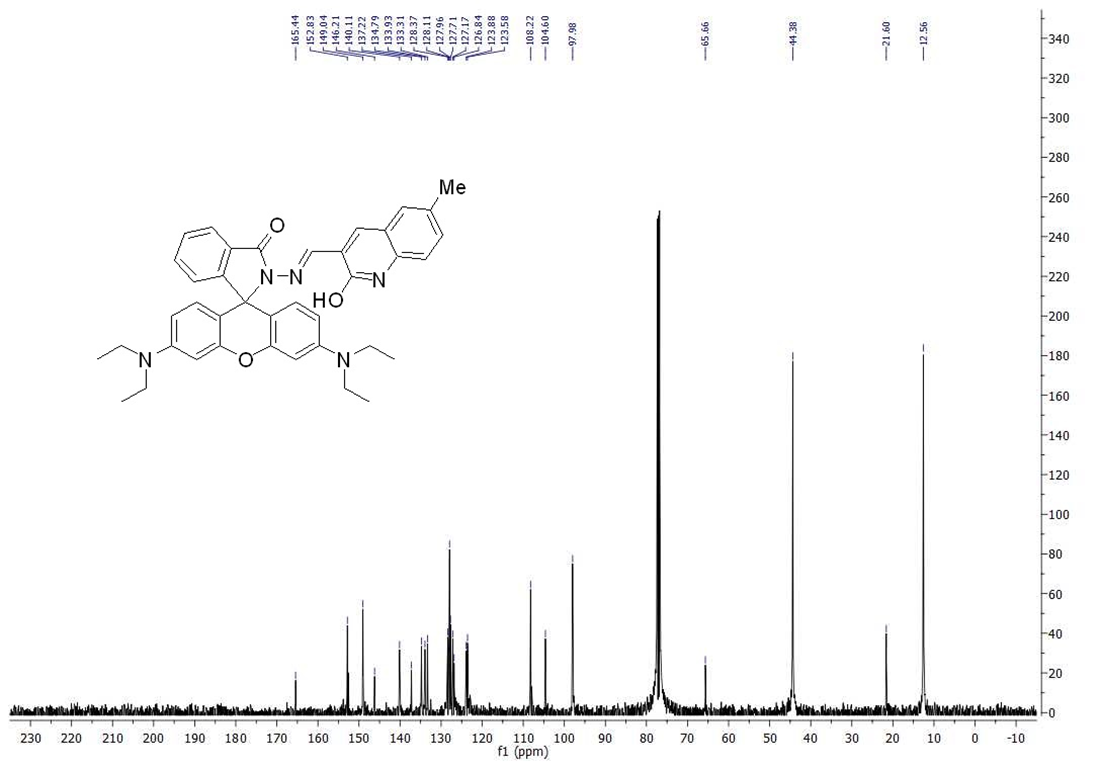


**Figure 8.** ^13^C NMR spectrum for 6


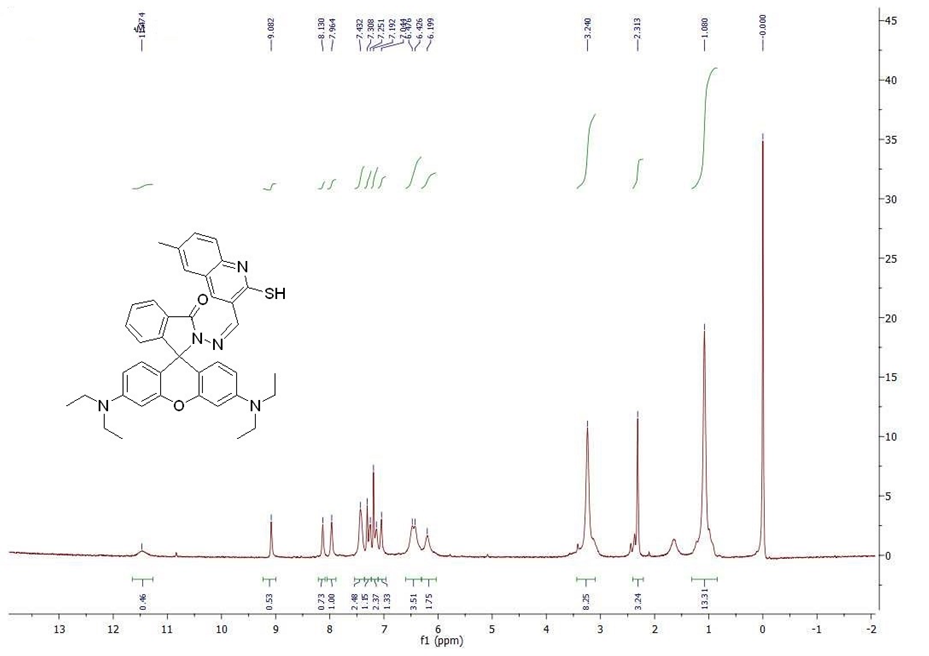


**Figure 9.** ^1^H NMR spectrum for 7a


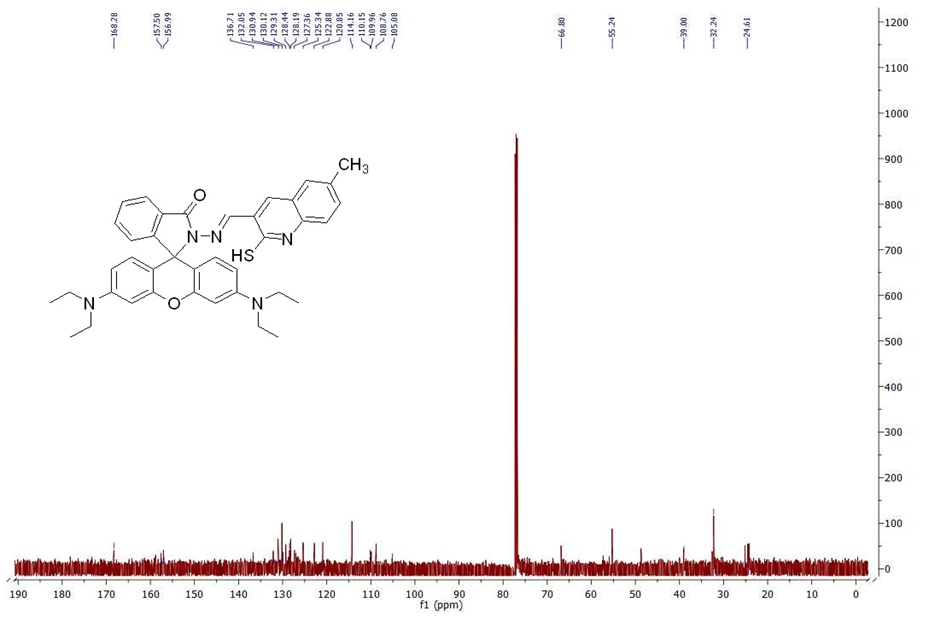


**Figure 10.** ^13^C NMR spectrum for 7a


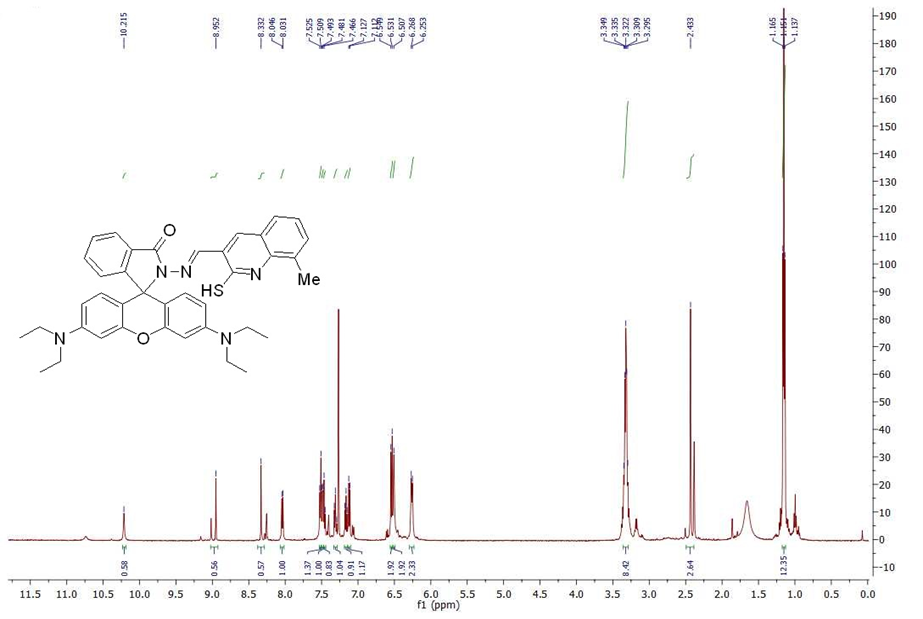


**Figure 11.** ^1^H NMR spectrum for 7b


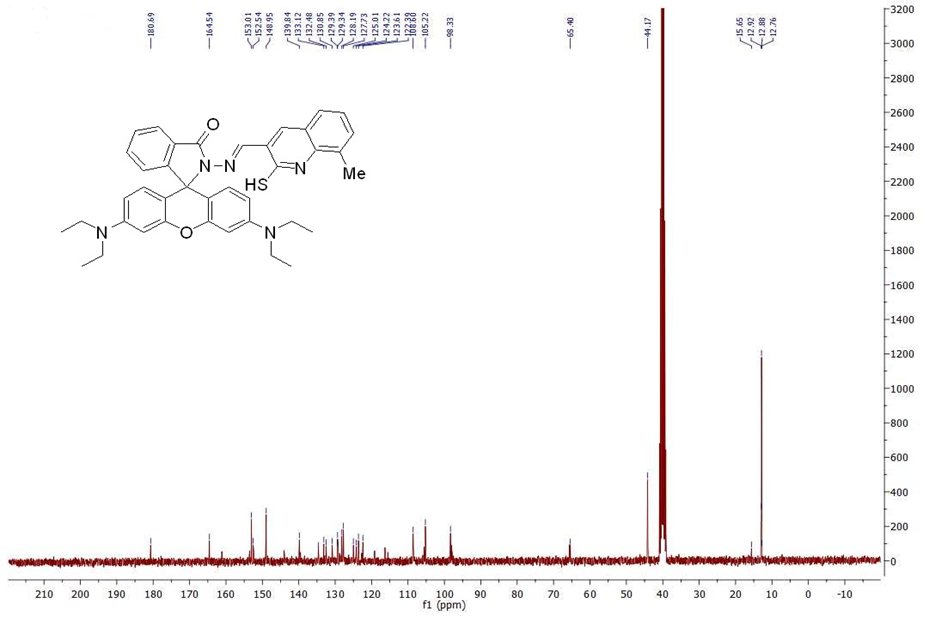


**Figure 12.** ^13^C NMR spectrum for 7b


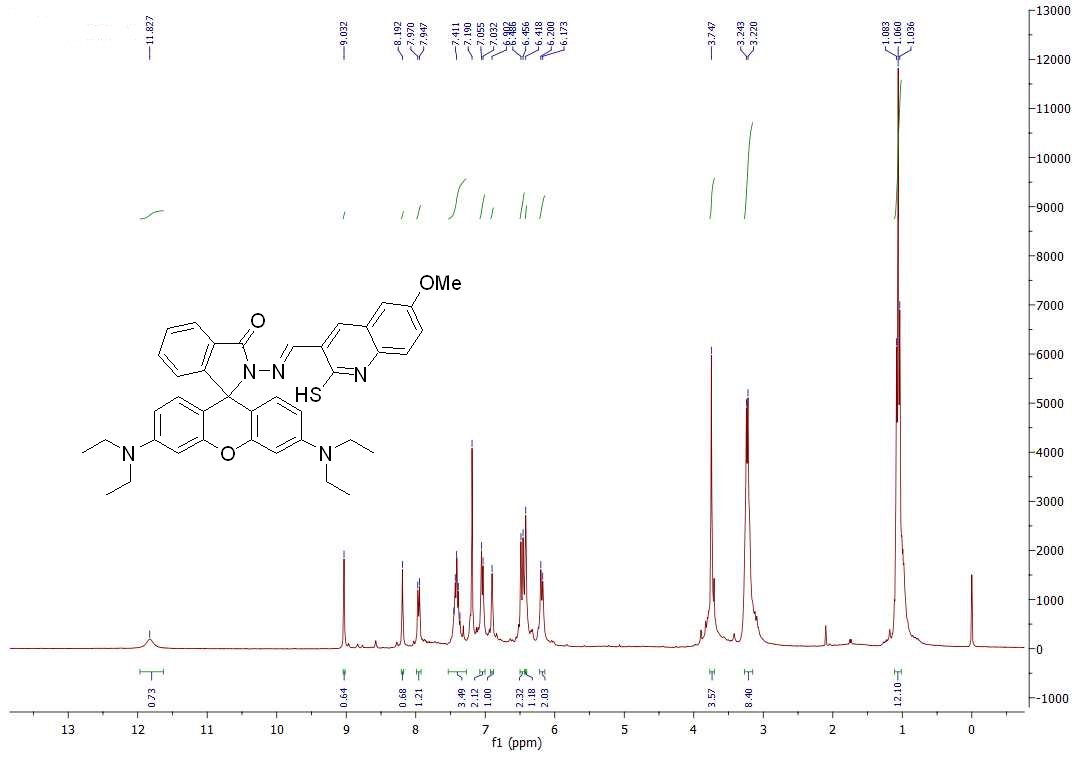


**Figure 13.** ^1^H NMR spectrum for 7c


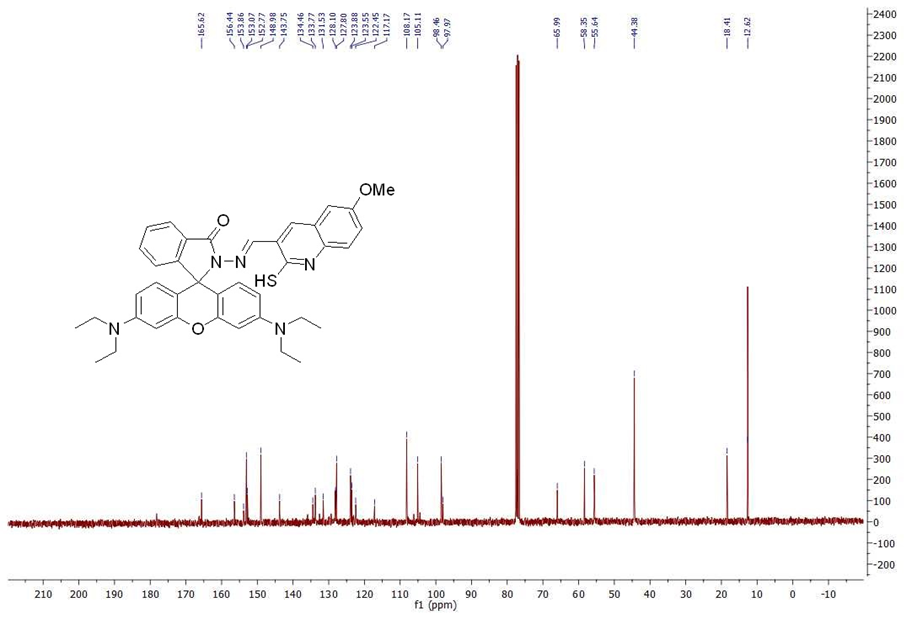


**Figure 14.** ^13^C NMR spectrum for 7c


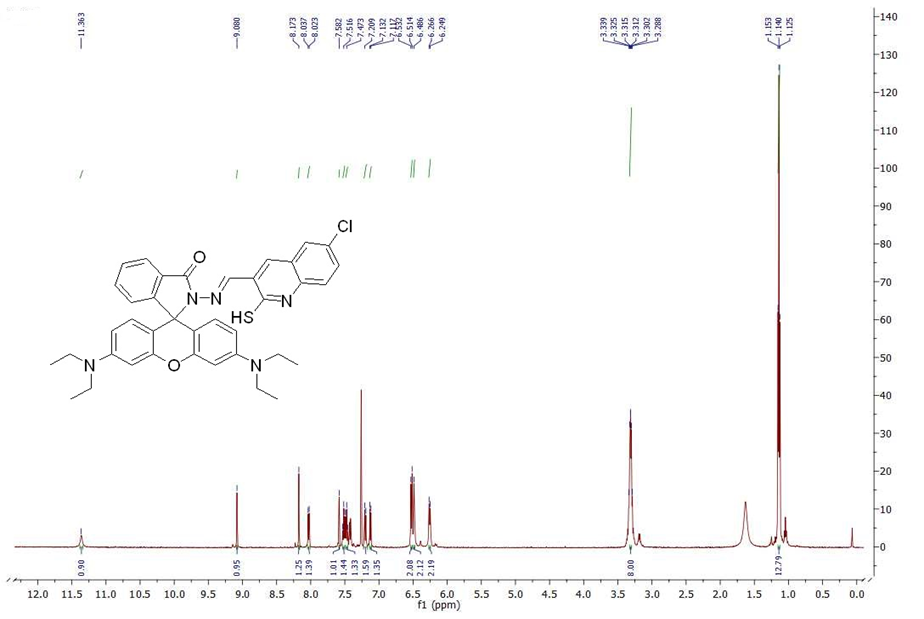


**Figure 15.** ^1^H NMR spectrum for 7d


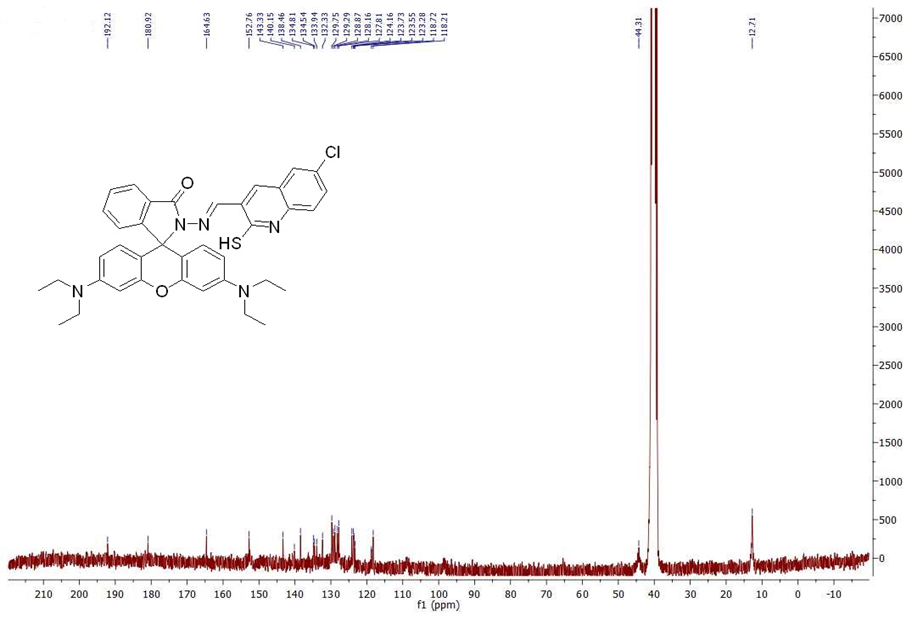


**Figure 16.** ^13^C NMR spectrum for 7d


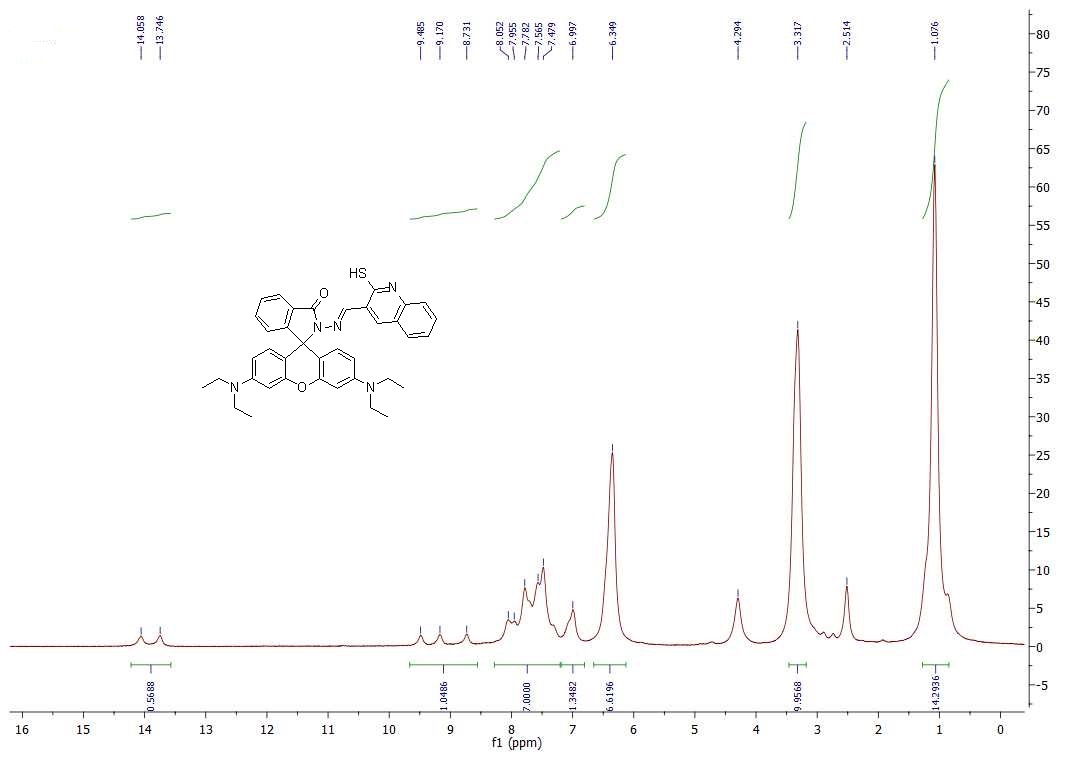


**Figure 17.** ^1^H NMR spectrum for 7e


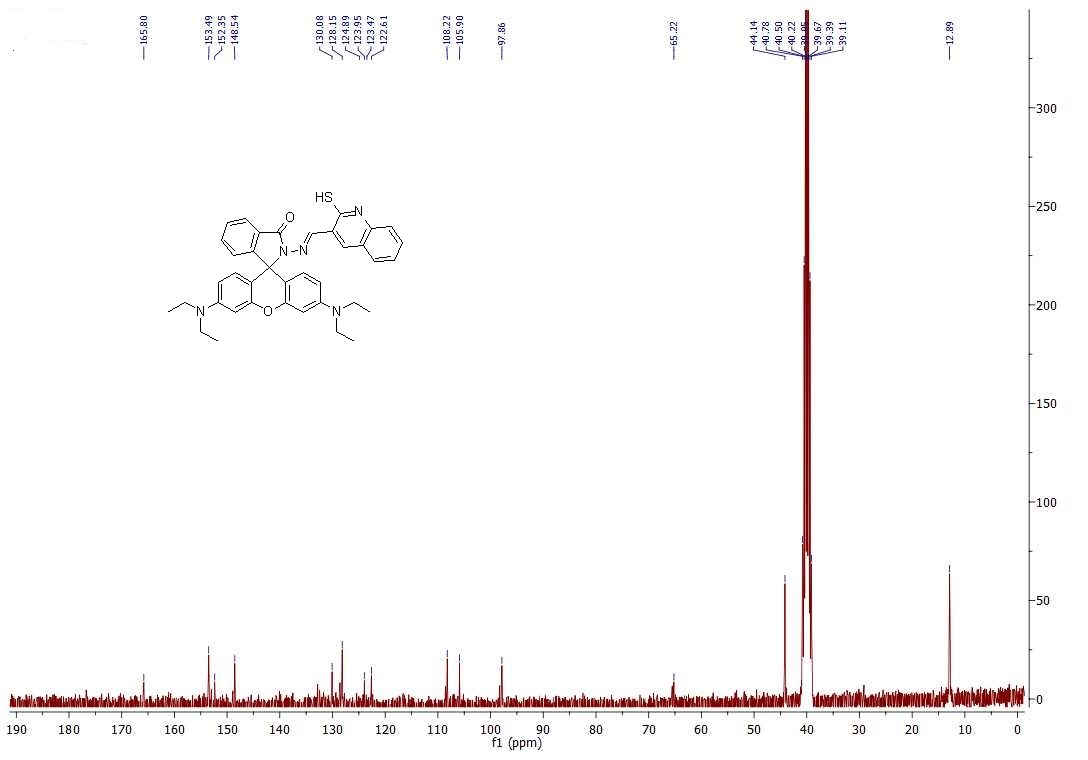


**Figure 18.** ^13^C NMR spectrum for 7e
